# Understanding Interpretations of and Responses to Childhood Fever in the Chikhwawa District of Malawi

**DOI:** 10.1371/journal.pone.0125439

**Published:** 2015-06-18

**Authors:** Victoria L. Ewing, Rachel Tolhurst, Andrew Kapinda, Miguel SanJoaquin, Dianne J. Terlouw, Esther Richards, David G. Lalloo

**Affiliations:** 1 Malawi-Liverpool-Wellcome Trust Clinical Research Programme, Blantyre, Malawi; 2 Liverpool School of Tropical Medicine, Liverpool, United Kingdom; 3 The World Bank, Phnom Penh, Cambodia; Tulane University School of Public Health and Tropical Medicine, UNITED STATES

## Abstract

**Background:**

Universal access to, and community uptake of malaria prevention and treatment strategies are critical to achieving current targets for malaria reduction. Each step in the treatment-seeking pathway must be considered in order to establish where opportunities for successful engagement and treatment occur. We describe local classifications of childhood febrile illnesses, present an overview of treatment-seeking, beginning with recognition of illness, and suggest how interventions could be used to target the barriers experienced.

**Methods:**

Qualitative data were collected between September 2010 and February 2011. A total of 12 Focus Group Discussions and 22 Critical Incident Interviews were conducted with primary caregivers who had reported a recent febrile episode for one of their children.

**Findings and Conclusion:**

The phrase ‘*kutentha thupi’*, or ‘hot body’ was used to describe fever, the most frequently mentioned causes of which were *malungo *(translated as ‘malaria’), *mauka*, *nyankhwa* and *(m)tsempho*. Differentiating the cause was challenging because these illnesses were described as having many similar non-specific symptoms, despite considerable differences in the perceived mechanisms of illness. *Malungo* was widely understood to be caused by mosquitoes. Commonly described symptoms included: fever, weakness, vomiting, diarrhoea and coughing. These symptoms matched well with the biomedical definition of malaria, although they also overlapped with symptoms of other illnesses in both the biomedical model and local illness classifications. In addition, *malungo* was used interchangeably to describe malaria and fever in general. Caregivers engaged in a three-phased approach to treatment seeking. Phase 1—Assessment; Phase 2—Seeking care outside the home; Phase 3—Evaluation of treatment response. Within this paper, the three-phased approach is explored to identify potential interventions to target barriers to appropriate treatment. Community engagement and health promotion, the provision of antimalarials at community level and better training health workers in the causes and treatment of non-malarial febrile illnesses may improve access to appropriate treatment and outcomes.

## Introduction

Universal access to and community uptake of malaria prevention and treatment strategies is critical to achieving current international targets for malaria reduction such as Millennium Development Goal 6 and the Global Malaria Action Plan to target the long-term eradication of malaria [1,2]. Artemisinin-based combination therapies (ACT) are highly efficacious, and prompt case management with ACTs is essential to reduce the global burden of malaria. Reviews of the literature on treatment-seeking show that children with most febrile illnesses receive some form of treatment, but that home treatment is often tried first [3–5]. The Malawi Demographic and Health (MDH) 2010 and National Malaria Indicator Surveys 2010 found that fewer than half (43.4% and 30.9% respectively) of recently febrile children under the age of 5 received an antimalarial drug [6,7]. While the introduction of rapid diagnostic tests for malaria will support appropriate diagnosis and treatment by health professionals, interventions to improve prompt, appropriate and effective treatment also require better understanding of the treatment-seeking behaviour of caretakers for sick children.

Studies have highlighted the importance of considering the step-by-step process of treatment-seeking in order to establish where opportunities for successful treatment are lost [8,9]. These studies often consider primary sources of care and sources of allopathic medicine only. However, individuals frequently utilise multiple sources of care, often starting with home treatments and moving on to health facility care [4,5]. Treatment-seeking begins with recognition of illness; perceived causes and appropriate responses to symptoms then determine what action, if any, is taken. Some studies have linked perceptions about the cause, prevention or treatment of malaria with ineffective prevention and treatment strategies [10–12], although individuals have been found to perceive illnesses as less severe in situations where treatment-seeking is more challenging, as a strategy to cope with the cost of illness [13]. McCombie highlights the importance of investigating local illness categories and the great variation between cultures [4]. New health information received by communities interacts with existing knowledge and ideas, and new concepts develop that may be quite different from the intended messages [14]. As a result of such syncretism, it is important that up-to-date studies are conducted, to ensure that appropriate messages are getting through. This is especially true after the introduction of new treatments. ACTs were introduced in Malawi in 2007, since when limited detailed qualitative work has been conducted to explore treatment-seeking behaviour.

Symptoms of malaria were known to overlap with symptoms of other illnesses in the local Malawian conceptualisation as well as in the biomedical model [15–17]. We therefore conducted an investigation to describe local classifications of childhood febrile illnesses. The results of the study were used to develop an overview of treatment-seeking, beginning with recognition of illness and considering utilisation of all and multiple sources of care. Barriers to effective treatment-seeking are discussed for each treatment-seeking phase.

## Methods

### Study site

This study was situated within the Chikhwawa district, in the southern region of Malawi. It was nested within a community-based trial investigating the programmatic implementation of ACTs in children below 5 years of age (Clinical Trial ID No: NCT010380632). Malaria is the leading cause of morbidity and mortality in children under five and pregnant women in Malawi, with an estimated six million cases annually [7]. The 2005 integrated household survey found that 52% of the population live beneath the upper poverty line, classed as poor and 22% live beneath the lower poverty line, considered ultra-poor [18]. The southern region holds the largest proportion (60%) of individuals living beneath the poverty line. The Malawi National Malaria Indicator Survey 2010 found that 39% of children under five years had had fever in the previous two weeks, with comparable results from the Chikhwawa district (37%) [7]. The majority of households in the Chikhwawa district depend on subsistence farming. This study was conducted in the Chikwawa district because of the high incidence of fever in children and the opportunities arising from the parent trial.

The Malawi health system is divided into three levels: a limited number of referral hospitals in major urban centres (tertiary level); district hospitals (secondary level); health centres and clinics (primary level). Within the Chikhwawa district, basic care is also provided at the community level by Community health workers (CHWs), known locally as Health Surveillance Assistants, who deliver treatment from health posts and conduct health education campaigns. First-line treatment of malaria switched from sulfadoxine-pyrimethamine to ACTs (artemether-lumefantrine; locally referred to as LA) in 2007. At the time of this study, ACTs were not provided by community health workers but could only be accessed in health centres.

### Study design

This qualitative study was conducted between September 2010 and February 2011 and included a series of Focus Group Discussions (FGDs) and Critical Incident Interviews (CIIs) (Table 1). Four local fieldworkers conducted the qualitative data collection in the local language (predominantly Chichewa, although Chisena was used in some villages). 12 FGDs were conducted with male and female community members in two rounds in two different subsets of the population. Separate FGDs were held with groups of younger and older women and men. Two male fieldworkers conducted FGDs with male participants and two female fieldworkers conducted FGDs with female participants. Critical Incident Interviews (CIIs) were conducted with primary caregivers of children who had recently experienced a febrile episode. 22 eligible individuals were initially identified and CIIs were conducted with each of them. At this point no new themes or ideas appeared to be arising and so no further CIIs were conducted. All the CIIs were conducted with female participants by one of the female fieldworkers.

**Table 1:** Qualitative methods.

Focus group discussions*	
Young women	4
Older women	4
Men	4
**Total Focus Group Discussions**	**12**
Critical incidence interviews	
Women whose child experienced fever and attended a formal health facility	12
Women whose child experienced fever and did not attend a formal health facility	10
**Total critical incidence interviews**	**22**

*8 to 10 participants per group

#### Focus Group Discussions

FGDs used early in data collection can help develop definitions of the topic being explored [19,20]. Six initial FGDs were used to gather information about community definitions of malaria and other causes of fever and norms around appropriate responses to each of the perceived causes. Repeat FGDs were conducted with each group to discuss the earlier findings and to clarify and probe further into the issues that had been identified.

Maximum variation purposive sampling was used to select FGD participants, ensuring that a range of perspectives was included [21,22]. FGDs were conducted in two village clusters, representing two diverse sub-sets of the population in the Chikhwawa district—one was a group of villages near-the-hospital (NTH), defined as within 5km of the district hospital and the other was a group of villages far-from-hospital (FFH). FFH villages selected for FGDs were defined as hard-to-reach by the Ministry of Health (MOH), on the basis of being more than 8km from a health facility or experiencing limited access due to the presence of geographic obstacles such as rivers or hills.

The fieldworkers were not sufficiently familiar with the local villages to select participants. Instead, members of Community Advisory Groups (CAGs) that had been set up as a point of contact between the researchers of the parent study and the community were requested to select individuals. Discussions held with CAGs during selection emphasised identifying ‘symbolically’ representative members of the community; criteria included selecting individuals with metal and straw roofs; those who lived nearer and further from the village centre; and those from different family groups. The team were aware of the likelihood that friends and relatives might be selected, however previously collected household wealth data suggested that within each sub-community, individuals were fairly homogenous in terms of possession ownership as a proxy for wealth [23]. Male and female FGD participants all had one or more children, choice of participants was not restricted by age of the child in order to ensure the views of older community members were captured. The younger women’s groups were restricted to married women and women with fewer than four children, as CAGs advised that unmarried young women may not have felt able to participate freely in groups of married women and women with more children would be considered experience which may have inhibited other women’s ability to contribute. Younger women were mainly in their early 20s. Identified individuals were visited in their homes by fieldworkers and invited to participant in the FGD. Fully informed written consent was obtained at this time and the participants informed of the place and time of the FGD.

#### Critical incident interviews

The initial and subsequent FGDs found that in most cases caregivers suspected non-resolving fevers to be malaria. Selection of CII participants therefore focused on the experiences of those whose child’s recent illness was likely to have been malaria. Participants were purposively selected from individuals who participated in a continuous (‘rolling’) Multiple Indicator Survey (MIS) during the period of qualitative data collection [24]. The MIS was conducted as part of the parent study to monitor malaria in the study population. A malaria rapid diagnostic test (RDT) was done for all surveyed children; those with a positive RDT who had not received antimalarials were treated, those with a positive RDT who had been treated within the two weeks preceding the survey were referred to hospital for further assessment. CIIs were conducted with two groups of MIS participants: primary caregivers who had not attended a formal health facility for their child’s recent illness, if the child tested positive for malaria during the survey and fever had been recognised for more than 24 hours; and primary caregivers whose children had received and completed malaria treatment obtained from a health facility within the two weeks preceding the survey. Individuals in both groups were considered to have had probable malaria enabling comparison of treatment-seeking between those who had attended a formal health facility and those who had not. CII participants were equally divided into those living less-than-15-minutes (NTH) and greater-than-15-minute walk from a health centre (FFH). All MIS survey participants had been informed that a small number of people would be selected to participate in a follow-up interview and asked for permission to be contacted if they were selected. Eligible individuals were visited in their homes by fieldworkers between the day after and 14 days after treatment should have been completed. Fully informed written consent was obtained at this time and a suitable time and place was arranged for the interview.

#### Topic guides

Topic guides were used during FGDs and CIIs to ensure that relevant topics were covered, however these were used flexibly to enable a sense of relaxed dialogue and probing. FGD topic guides were used to guide the discussions through three main topics: initially perceived causes of childhood fever were explored—caregivers were asked to describe each and any methods used to differentiate the different causes. Secondly participants were asked to describe appropriate responses to each of the different perceived causes of fever. Finally the treatment-seeking process was explored including discussion about which individuals were involved and the challenges experienced at each stage. When discussing fever broadly, the fieldworkers were instructed to use the literal translation, *kutenta thupi*, or hot body. Short vignettes were provided and participants asked to role-play and discuss different treatment-seeking scenarios.

CIIs initially explored the household structure and the division of roles and responsibilities. Individuals were then guided to provide a narrative account of their child’s illness from recognition of illness to time of recovery or interview. This included perceptions of the illness; whether and how these changed through the course of the illness; interactions with other people; and the sources of care utilised. Draft topic guides were reviewed and refined with the fieldworkers before and after piloting.

### Training and Quality assurance

Fieldworkers undertook a series of training sessions in qualitative research methods, with a specific focus on conducting CIIs and FGDs. Training sessions also explored issues around treatment-seeking for childhood fever. Voice recordings of each IDI were reviewed on return from the field; translated transcripts of the pilot FGDs were discussed in detail, highlighting specific areas of good practice and those requiring further development.

Quality checking of transcribed data was conducted by a second transcriber who reviewed 10% of the transcripts against the audio file. The quality assurance of translation was conducted through back-translation by a second translator. Initially small sections were back-translated with further back-translation if discrepancies were found. The translators and transcribers met with the PI regularly to review transcripts and to discuss any issues arising.

### Data analysis

The team met after each qualitative data collection to review transcripts and discuss emerging themes. Data analysis was based on a framework approach [25]. The CIIs and FGDs were recorded, transcribed and translated into English then imported into the qualitative analysis software QSR NVivo 9 for management and analysis. All transcripts were read through and summaries written. A broad index was developed during coding of the first two FGDs and CIIs (by VE in discussion with AK and RT), based on themes emerging from the data. The index had the following main categories: Accessibility; Illness Typology; Diagnosis; Disease Outcome; Finance; Interactions; Treatment type; Narrative; People; Perception; Recognition; Roles and responsibilities; Service Quality; Signs and Symptoms; Steps in treatment-seeking pathway. The index was then applied to code all data and iteratively refined throughout coding (VE and AK under guidance from RT). Data were charted by summarising and comparing data coded under different indexes and across different categories of participants, and these charts were used to develop descriptive analyses and explanations (VE, RT and ER).

### Ethics statement

Ethical approval was obtained from the College of Medicine Research and Ethics Committee, Malawi (P.04/09/783 and P.10/08/707) and the Liverpool School of Tropical Medicine (09.07). Additionally permission was granted from village leaders. Sensitisation campaigns were conducted as part of the parent study to inform the communities about all aspects of the trial and affiliated studies. Fully informed written consent was obtained from all participants in the local language.

## Results

FGD and CII participants provided a range of interpretations of febrile illness, resulting in different treatment-seeking approaches. Specific responses to febrile illness were influenced by the context of the individual illness episode. Important factors included: geographic accessibility of formal health facilities; costs; time of day of illness onset; season; and intra-household dynamics, such as the decision-making authority of the primary caregiver, which was found to be particularly low for junior women in remote areas. Although specific responses varied according to illness context, an overall three-phased pattern of treatment-seeking was identified, and is described below. Findings are organised into two sections: the first describes local interpretations of febrile illness; the second presents a description of three-phase treatment-seeking derived from the qualitative data.

### Interpretations of febrile illness

Interpretation of febrile illness were explored in detail during FGDs, however the findings were supported by discussions in CIIs. In the local language, the phrase ‘*kutentha thupi’*, or ‘hot body’ was used to describe fever. A number of illnesses were said to cause this. The most frequently mentioned were *malungo*, *mauka* (also *masungu*), *nyankhwa* and *(m)tsempho*. The symptoms of these illnesses were described as similar, despite considerable difference in perceived causes.

#### *Malungo* (Malaria)

*Malungo* (translated as ‘malaria’) was widely understood to be caused by mosquitoes and to a lesser extent, by the weather (exposure to hot sun), hunger and lack of hygiene. In one FGD, poverty was emphasised as causing malaria, mainly through the mechanism of causing people to work hard in the sun with little food. *Malungo* was considered to consist of a complex of symptoms similar to the clinical definition of malaria, although these symptoms were non-specific and also overlapped with other perceived causes of illness described below. Commonly mentioned symptoms included: fever, weakness, vomiting, diarrhoea and coughing. Less frequently mentioned symptoms included: sneezing, stomach ache, fainting, shivering, gnawing teeth, hallucinations, yellow or red eyes, headache, loss of appetite and aching body. The terms *malungo* and *kutentha thupi* were not always differentiated when used to describe febrile illnesses; at times ‘*malungo*’ was used to refer simply to fever. Despite this, the presence of fever (*kutentha thupi*) alone was not sufficient for the illness to be considered malaria requiring antimalarial treatment.

Collapsing was reported in all the FGDs to be a sign of severe malaria. Collapsing was not considered a symptom of *mauka*, *nyankhwa* or *tsempho and* in one FGD was identified as a symptom which could help differentiate between these illnesses and malaria. Older woman in villages FFH mentioned cerebral malaria (*malungo ali mu ubongo*) in FGDs. They said it caused children to collapse, hallucinate and “talk nonsense”. However, collapsing, even with fever, was not always interpreted as cerebral malaria. One CII participant explained that they initially thought their child, who had fever and collapsed, did not have malaria, but *mfayi* (chisena word translated as epilepsy):

**Respondent:** *No*, *he had fever all night till morning and in the morning*, *he collapsed…*

**Interviewer: *…*** *What disease did you think it was*?

**Respondent:** *I thought it was epilepsy…*

(CII, attended a health facility, FFH, 04/11/10)

Another CII participant thought their child’s fever and collapsing indicated *juni*, an illness the participant likened to tetanus (*kafumbata*). Both these CII participants had attended a health facility for their child’s illness.

#### *Mauka* (also *masungu*) and *nyankhwa*

*Mauka* (also *masungu*) and *nyankhwa* were also identified across all FGDs as causing childhood fever. They were considered similar to each other in aetiology and presentation and there were overlaps with *malungo*: vomiting and weakness were frequently described as signs of *mauka*, and both *mauka* and *nyankhwa* were said to cause diarrhoea. These illnesses were described as emanating from the mother, who experiences flat rash-like sores (*mauka*) or growths (*nyankhwa*) in or around their vagina or anus (but no fever), and passed onto the child through breastfeeding.

#### Tsempho

Some symptoms of *Tsempho* overlapped with those of *malungo*, *mauka* and *nyankhwa*. Fever, coughing, weakness and diarrhoea were frequently reported. Other, progressive symptoms of *tsempho* were more distinct—the child becomes thin, stunted, swollen and anaemic. The breaking of various, complex taboos were believed to cause *tsempho*, summarised well in the following quote:

*Tsempho occurs when we don’t follow the customs and traditions as taught by our elders in our homes*. *Our parents teach us certain things that we must do in our houses when a man*, *a woman and their child share the same room*.

(Younger women's FGD, FFH, 08/10/10).

Participants described a number of taboos which revolved around sexual practices and the positioning of the child in the bed [on the mat].

#### Other perceived causes of childhood fever

Participants described ‘ordinary’ fever, not attributed to any particular disease and requiring nothing more than shop-bought treatment. Causes included the weather (too hot or too cold), excessive crying, and teething. Parents considered not only the fever, but other signs that would help them ascertain its cause. Flu, cough and measles were also understood to cause fever. Witchcraft (*ufiti*) was thought to be responsible for childhood fever, as a mechanism causing sickness.

### Responses to childhood fever

**Fig 1:**
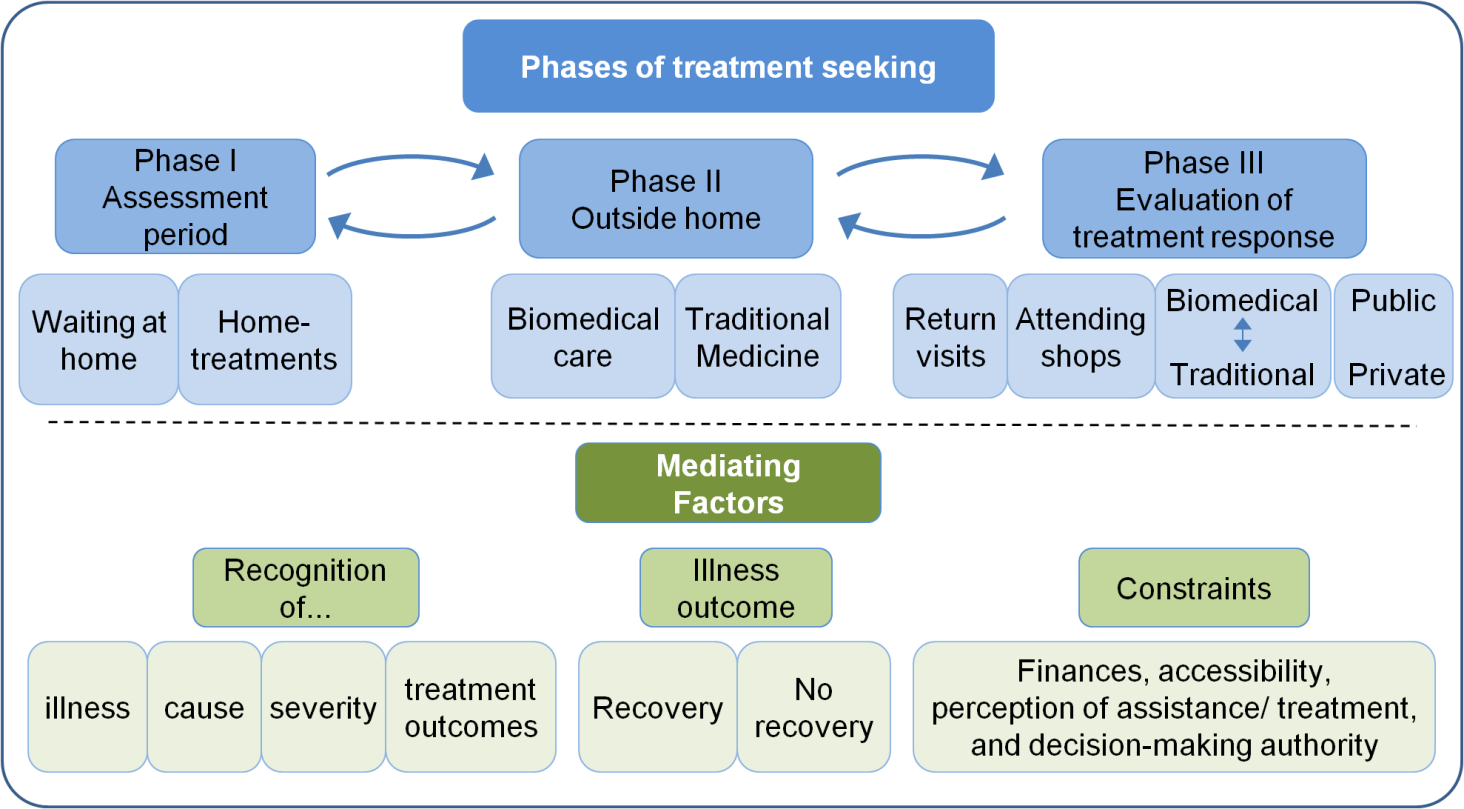
Diagrammatic representation of the treatment-seeking process.

The analysis of the qualitative data led to the development of an overview of the treatment-seeking process in three phases: Phase 1—The assessment phase; Phase 2—Seeking care outside the home; Phase 3—Evaluation of treatment response (Fig 1).

### Phase 1: Assessment period

The usual first step after recognition of illness was to assess its severity. Participants frequently differentiated between mild forms of illness, which could be treated using home remedies, and more serious forms requiring hospital assistance:

*Yes*, *the extent of the sickness*. *If he is very sick*, *we rush to the hospital because a child can be sick and be able to play*. *Yet another child would be too sick to eat or play; he just sleeps*. *It won’t help to buy drugs from a shop; we’d better take the child to the hospital*.

(Younger women’s FGD, NTH, 22/01/11)

In most cases illnesses were described as initially mild; caregivers observed the child to assess whether the illness became more severe or resolved. Lack of perceived severity was the most common reason why CII participants whose child had fever and had tested positive for malaria by the MIS team had not attended a health facility; in most cases the fact that the child was still playing was taken as a sign that illness did not require hospital intervention.

Immediate recognition of an illness as serious was rare; however caregivers stressed that in situations where an illness was recognised as serious they would go straight to the health facility. The process of assessing illness severity usually involved a ‘wait and see’ approach, most often combined with some form of action, such as using purchased drugs or bathing the child in an attempt to cool them. When symptoms fail to resolve or worsen, hospital care was sought:

W*e try to see*, *as I said before*, *whether or not it is real malungo*. *When he starts in the evening*, *we wait till morning to see how he will spend the night and in the morning when we see that the fever is still there*, *we will try some small drugs like children’s cafenol or panado so that if it is ordinary*, *simple fever*, *then the drugs will work and the fever will cease*. *When we try the children’s cafenol and the fever does not stop it or it increases*, *then we think of taking the child to the hospital*.

(Men’s FGD, FFH, 07/10/10)

As described in the above quote, milder, resolving fevers were not necessarily categorised as a particular ‘disease’, but were often referred to as ‘simple fever’. In most instances, lack of recovery after the initial period of assessment was described alongside symptoms indicative of *malungo*, and this combination was considered evidence that malaria was the cause, prompting attendance at the hospital. Caregivers explained that it usually took between two and five days, sometimes longer, to recognise their child has *malungo*. Several CII participants who had not attended a health facility said they had planned to do so, but had not yet done so when the MIS team arrived. This period of waiting meant that the illness could be quite serious by the time they attended hospital. Other reasons for not attending a health facility until the illness was serious included the difficulties with recognising illness until it had progressed, and rapid development of illness such that it became serious before the caregiver had a chance to respond.

External constraints also influenced decisions by caregivers. Shop-bought drugs were sometimes used while caregivers established illness severity and the need for other care. However at times shop-bought drugs were used because of difficulties in accessing health care, rather than strategically to assess illness severity. A number of references were made in both FGDs and CIIs to using shop-bought drugs at night-time to control the fever until the child could be taken to a health facility. Caregivers did not always appear satisfied with the use of shop-bought drugs; comments about the use of expired shop-bought drugs were made in male FGDs in NTH and FFH villages:

**Respondent 1:** *Especially at the grocer’s in our villages*, *we are just given drugs which perhaps expired long ago*, *and sometimes these drugs are kept in hot places*, *not good for drugs*.

**Interviewer:** *So we just buy*?

**All:** *Yes*!!

**Respondent 2:** *We just buy them*. *By the time we go to the hospital*, *it means we have failed*. *Let’s be honest*, *before we reach the hospital*, *we try all ways available and when we fail*, *it’s when we go to the hospital*.

(Men’s FGD, FFH, 07/10/10)

### Phase 2: Seeking care outside the home

If symptoms did not resolve after the assessment period, individuals moved to Phase 2, meaning that individuals had to decide which source of care to attend outside the home. All of the symptoms associated with *malungo* were said to prompt caregivers to seek formal healthcare (public or private health facility, or CHW).

A *sing’anga* (traditional healer—*asing’anga* plural) was perceived to be the only place to receive treatment for illness caused by witchcraft, *tsempho*, *nyankhwa*, and usually mauka. A*sing’anga* were thought to be the only ones capable of identifying witchcraft as the cause. Despite this, most FGD participants stated that if the illness did not resolve after the assessment period, then care would be sought from a formal health facility initially. Reasons for doing this rather than visiting a traditional healer included the potential for malaria to progress rapidly leading to death; difficulty in recognising traditional causes of illness; and the importance of biomedical diagnosis:

*But first*, *it is important to rush to the hospital before going to the traditional healer because the traditional healer may tell lies since he doesn’t have any machine for diagnosis*, *he will just give you lots of medicine and that may create many problems*. *That’s why it is really important to first of all rush to the hospital for diagnosis*.

(Men's FGD, NTH, 06/10/10)

However, participants described situations where it was possible to recognise that an illness required assistance from the *sing’anga* in the first instance, rather than from a health facility. The main example of this was when the child suffers fever and the mother ‘an itch’ at the same time (*mauka* and *nyankhwa*).

### Phase 3: Evaluation of treatment response

Caregivers relied on lack of resolution, or increased severity of symptoms as indicators of treatment failure and described reduced fever, increased strength, eating and playing as signs of recovery. The treatment-seeking process was not linear and caregivers described how after attending a source of care outside the home, they would then assess whether treatment had worked.

Some members in all FGDs indicated they usually returned to the health facility before trying other sources. These participants emphasised that they might make several trips to the hospital before searching out other methods. This was supported by the findings of CIIs: three participants had made return visits to the hospital. A minority of FGD participants mentioned switching from public to private health facilities if treatment was unsuccessful. Reasons for returning to the hospital rather than seeking care from elsewhere included: getting another opinion; hospital treatment being free; showing others that you did all you could; getting another drug; and convincing doctors of the severity of the illness. A minority of FGD participants suggested that they would switch to a traditional healer rather than make a return visit. Reasons for not returning to the health facility included: receiving a negative malaria test at the first visit, which some considered to indicate a traditional illness; fear of receiving the same drug (LA) again; and no improvement upon completion of medication, which was felt to indicate a traditional cause. Occasionally, participants described visiting traditional healers in-between health facility visits. Repeat visits were also described for shops and traditional healers.

Attendance at a second source of care was not necessarily a sign of treatment failure. CII and FGD participants explained that they were frequently advised to obtain shop-bought paracetamol to take alongside LA. In some cases individuals outlined their belief that LA cannot work properly unless combined with paracetamol.

Individuals combined observed symptoms with treatment responses in establishing their final perceived causes of illness, through a process of reinterpretation, or trial and error. Illness cause was recognised retrospectively by successful treatment. When hospital treatment was unsuccessful, individuals began to doubt malaria as the cause. Recognition of *mauka* was an example of this reinterpretation process frequently described in FGDs and also in one CII. If the mother had symptoms, the next stage was to treat her, either with biomedical or traditional treatment. Treatment failure was described as being particularly important in recognising witchcraft and *tsempho*, as these illnesses were not considered to be readily identifiable during the initial stages of illness. Treatment failure leads to the suspicion that somebody is to blame for the child's illness, either through their unfaithfulness (a parent) or through engaging in witchcraft (usually a relative). However successful treatment by the traditional healer was the ultimate proof of cause. Witchcraft and *tsempho* were not described in CIIs, perhaps because these were considered later in the course of the illness and the CIIs only captured recent illness.

The response to non-resolving fever sometimes involved re-assessing the cause of illness and switching providers. However caregivers did not necessarily switch providers just because their perception of the cause had changed. The order of treatment options was influenced by attempts to minimise costs, as well as which source of care was perceived to be most suitable. At times a source of care was used because they had given up on the previous one, rather than because they had reinterpreted the illness. The following quote demonstrates how perceived lack of treatment options at health facilities results in individuals’ pursuing alternative options:

*We go again and when we are given LA again and there is no change then we make plans to see other people to help us*. *At the hospital we are told that the only available drug is LA*, *so*, *as beggars*, *we do not have anything to say*.

(Men’s FGD, NTH, 17/01/11)

Some Individuals combined traditional and biomedical sources of care and did not categorise all illnesses as only having a traditional or biomedical cause, illustrated by the following quote:

*When the mother has mauka removed*, *what remains is just ordinary malaria that is treated using LA and if the child doesn’t recover*, *all the mother does*, *is to go to the hospital for change of treatment or to get another dosage*.

(Younger women’s FGD, NTH, 22/01/11)

Other examples included the belief that children may require hospital treatment for anaemia after receiving traditional treatment for *tsempho*: and the suggestion that biomedical researchers should do more to develop cures for illnesses with traditional names (male FGD).

There was disagreement among the participants about the appropriate treatment of mauka; some argued it could only be treated by a traditional healer, while others stated that it could be treated at the hospital. The following quote demonstrates the blurring of boundaries between biomedical and traditional theories, and how healthcare workers’ interpretation and management of childhood fever may further complicate matters:

*…at the completion of the dosage*, *we find that there is no improvement*. *Usually*, *we go back to the hospital and explain that the medicine has not been effective*. *The doctors then tell us that there is nothing wrong with the child and usually they ask if the mother doesn’t feel itching in the private parts and they suggest a certain sickness other than mauka and so they administer some pills to be applied in the private parts*.

(Younger women’s FGD, NTH, 06/10/11)

This support for traditional beliefs by some biomedical personnel was also shown by references to biomedical staff referring individuals to traditional healers.

Treatment failure did not always lead to reconsidering the cause of illness. Participants explained that illnesses caused by witchcraft (*ufiti)* may not be successfully treated by a traditional healer if the traditional healer is a fake, or if the spirits decide the child will not recover. In this way *ufiti*, in particular, may provide a kind of ‘fall-back’ explanation for unsuccessful treatment.

Caregivers also had to decide when further visits are no longer necessary. In a number of FGDs individuals stated that if they had been to the hospital with no success and then the traditional healer had failed, then they would give up:

**Interviewer:** *So when you go to the hospital for the first time and it doesn’t help*, *you go again and it fails again-*

**Respondent:** *Then we go to the traditional healer*.

**Interviewer:** *You go to the traditional healer*, *and when he fails*?

**Respondent:** *We become stranded*, *we really don’t have a solution*.

**Interviewer:** *Where do you go*?

**Respondent:** *Whatever happens*, *we give in (Laughter)*.

*(Men’s FGD*, *FFH*, *07/10/10)*

A few FGD and CII participants described turning to prayer or depending on God, either during treatment, or following failure of both biomedical and traditional sources of care. These references seem in contrast to the idea of ‘giving-up’; rather depending on God was considered an active process that gives hope:

*No*. *I wanted to just add that we do not just give up*, *but we commit everything in the hands of God*, *whether or not we sinned against him*. *We expect anything to happen*.

(Men’s FGD, NTH, 17/01/11)

Although severe illness was not investigated in detail it was notable that despite probing into treatment failure, there were few references to death. *Mauka*, *tsempho* and *ufiti* were described as potentially leading to death and in two of the FGDs (both with younger women) individuals stated that death may result from attempting to treat these illnesses at the hospital. However delays caused by attending a traditional healer were described in FGDs and CIIs as potentially leading to death or severe illness if the child had severe malaria:

*Whereas the witchdoctor will tell you to cut incisions all over the body of the child; in fact you just delay yourself and if the child has malaria in the brain*, *you might as well lose the child*

(CII, attended a health facility, FFH, 06/01/11)

## Discussion

**Fig 2:**
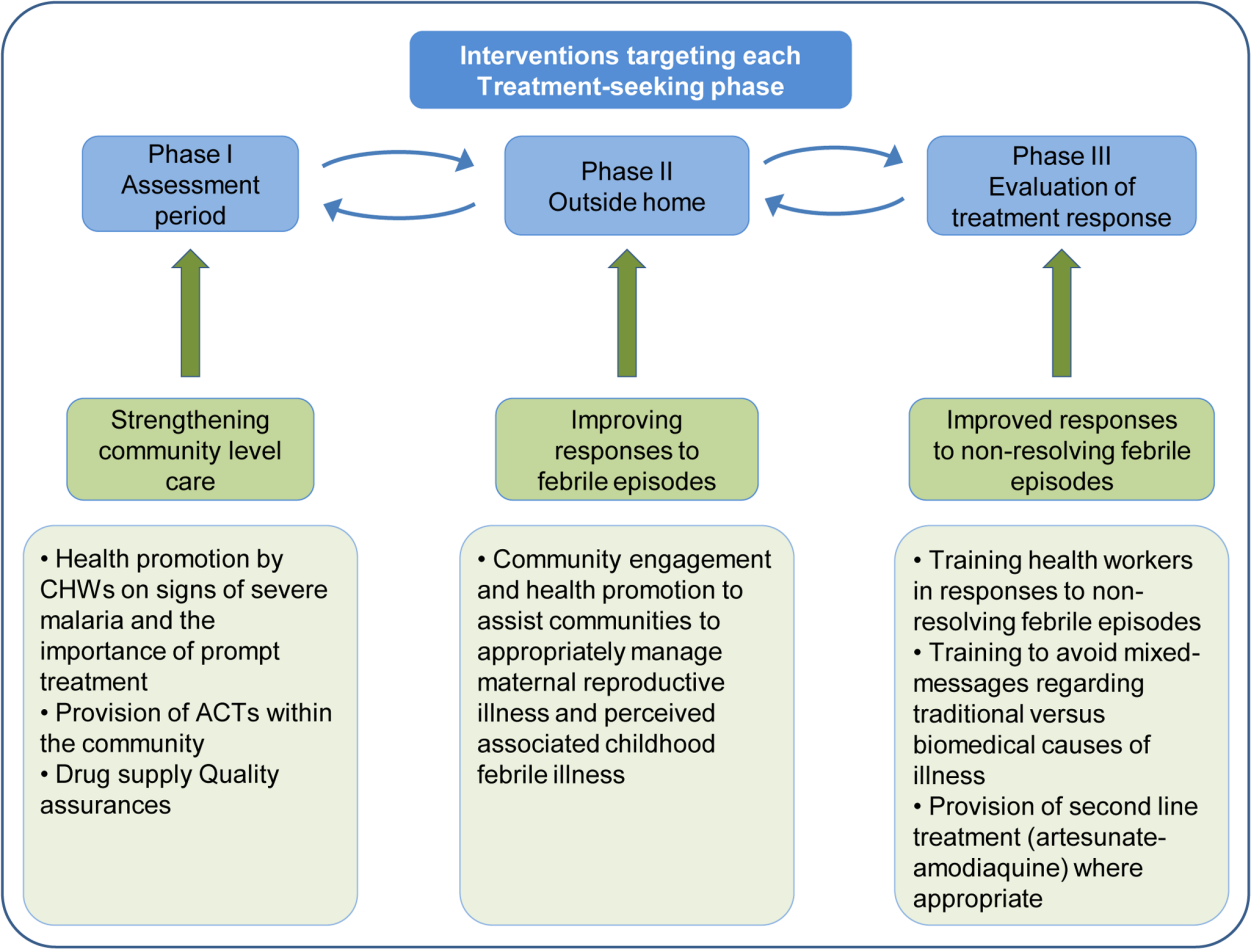
Interventions targeting each treatment-seeking phase.

The treatment-seeking process has previously been described as involving an initial generalised response to manage mild febrile illness, proceeding to decision-making based on treatment outcomes [5]. This study expands on this and findings were used to develop an overview of the treatment-seeking process in which three phases were defined: Phase 1 –the assessment period; Phase 2 –seeking care outside the home; and Phase 3 –evaluation of treatment response. Our increased understanding of this process may allow us to develop interventions that target each step in the treatment pathway (Fig 2).

Treatment-seeking was largely determined by knowledge of illness and interpretations of fever, as shown previously in Tanzania [26]. We found that descriptions of the symptoms of *malungo* were similar to those predictive of malaria in the biomedical model [15]. However interpretation of the term *malungo* was complex: as previously described in Malawi, it was used to describe fever broadly [27–30], though fever alone (*kutenta thupi*) was not always interpreted as *malungo*. This supports previous findings from Malawi the combined terms *malungo* and *kutentha thupi* better predicted the presence of biomedical malaria than each alone [31]. The need for health facility care for fevers that did not resolve during the assessment period was understood. However fever, even when combined with other symptoms of *malungo*, did not guarantee health facility attendance—perceived severity of illness was the key factor influencing treatment. Community-based health education and promotion programmes designed to engage with all those involved in treatment-seeking or provision of care within communities, including shop-keepers and traditional healers, may help to improve phase 1 decision-making.

The context of the individual illness episode was highly important; participants described how shop-bought drugs may be used strategically to ‘buy time’ until it is possible to attend a health facility. They were also said to be used even when perceived to be unsuitable, in order to avoid the challenges associated with health facility attendance. Distance, direct and indirect costs, and household related barriers have been widely described as inhibiting health facility attendance [4,5,32–34]. Distance and costs are important determinants of health facility utilisation within the Chikhwawa district and geographical constraints were seen to influence decision-making, despite good understanding of the signs of malaria and appropriate treatment [23]. In addition a study conducted in Burkina Faso found individuals perceived illness to be less severe during the wet season, when communities experienced increased demands of farming, highlighting the complex way in which multiple determinants of treatment-seeking interact [13,35]. Inappropriate sources of care will continue to be used unless constraints experienced during Phase 1 are reduced. Bringing care nearer to individual’s homes by adding quality assured ACTs to the package of care delivered by CHWs at community level, may improve access to appropriate treatment.

In common with previous studies, the majority of individuals reported actively seeking care for their child's febrile illness only after initial assessment. Public health facilities were the most frequently reported first source of care during phase 2. Health facilities have been found to be more frequently used in the first instance in situations where it is difficult to obtain antimalarials through informal sources and where treatment from public health facilities is free [5], both of which are true in the Chikhwawa district of Malawi. A previous Malawian study found the majority of individuals sought informal sources of care in the first instance, with many switching to formal sources in the second instance [36]. This difference may reflect successful changes in drug policy; LA was introduced as first-line treatment for malaria in Malawi between the two studies. At the time of the previous study, SP, the first-line antimalarial was, and continues to be, freely available from shops. Since the switch to ACT drugs, careful drug control has ensured that these highly efficacious drugs are not readily available from shops or small retail outlets in rural areas. The greater use of health facilities in the first instance is encouraging and may reflect positive perceptions of LA and its lack of availability outside of the formal health sector. However, it may also mean longer delays before children receive appropriate antimalarial treatment. Traditional care was reported to be used initially in some cases, if the febrile illness had a perceived link to maternal reproductive illness (Mauka/Nyankhwa). This highlights an area requiring focused community-engagement and health promotion activities. Community-engagement can provide opportunities for community reflection on current practices and enable the community themselves to establish appropriate changes [37]. Involving influential decision-makers, such as grandmothers, has been shown to positively influence treatment-seeking behaviour [38]. Consideration should also be given to involving non-formal providers, such as traditional healers, who hold local importance in treating such traditional causes of illness [37].

As previously reported, traditional causes of illness were generally considered after repeated failed hospital visits [39,40]. Unsuccessful treatment, both at the health facility and by traditional healers was interpreted within a syncretic model (which blends biomedical and traditional concepts) [14]; caregivers utilised different sources of care for different elements of the illness. Health facility staff contributed to this blurring of boundaries by suggesting traditional causes and referring individuals to traditional healers. Participants also expressed frustration with receiving repeated doses of LA and the lack of an alternative treatment. Such repeat visits place considerable economic burdens on households [41,42]. Re-treatment of children who return within 2 weeks of a previous treatment with first line antimalarials is common, but clearly needs to be considered urgently, both from a policy perspective and to improve phase 3 outcomes and support appropriate treatment-seeking. The potential role of health workers in communicating accurate information about the causes of, and appropriate responses to non-resolving fever has been previously highlighted [40] and has become more important with the increasing recognition that many childhood fevers are not due to malaria [43,44]. Within the Malawian context, health workers’ knowledge of these topics must first be strengthened. This should include training in the causes and treatment of non-malarial febrile illnesses and the consideration of underlying chronic conditions. It may also include the provision of a second line antimalarial if appropriate.

### Study limitations

The qualitative data collection focused on gathering women's views since women in this context tend to conduct the majority of child-rearing duties and were considered to have greater first-hand experience of the treatment-seeking process. Men in similar settings have been seen to be important decision makers within households [5,45,46] and therefore FGDs were also conducted with men. However the more limited data collection among this group may mean that male views and experiences are not as clearly expressed or represented. Additional sampling biases were present: FGD groups were selected by Community Advisory Groups set up as part of the parent study, and individuals selected may have represented more elite members of the community. However selection of villages for FGDs ensured both remote, more deprived communities were represented alongside the more central, wealthier villages and levels of wealth were known to be fairly homogenous within these sub-groups. Selection of individuals for CIIs did not include any children admitted as inpatients at the time of the MIS and therefore experiences of more severe illnesses may not have been fully captured. Selection of CII participants also focused on those whose child had recent probable malaria so experiences of non-malarial febrile illnesses may not have been fully represented.

Since this study was undertaken, Community Health Workers in hard-to-reach areas of the Chikhwawa district have increased the services that they provide and ACTs are now available at rural health posts. These are provided without diagnostic support. This could clearly have a potential benefit in reducing the waiting time during the assessment period and lead to more rapid treatment of malaria. However, as many childhood fevers are not due to malaria, it is unclear how a failure to respond to treatment will alter the population’s perception of formal health care and contribute to promoting initial alternative sources of care.

## Conclusion

The three-phased approach to treatment-seeking demonstrates the importance of considering the entire treatment-seeking pathway from initial assessment of illness severity, including attendance at non-formal sources of care and responses to non-resolving fever both by caregivers and health facility staff. Strengthening of community level care is required to improve the accessibility of services. This should be combined with focused community engagement and health promotion programmes that engage those involved in decision-making and treatment-provision at each phase of the treatment-seeking process.
